# Common variable immunodeficiency with granulomatous-lymphocytic interstitial lung disease and preceding neurological involvement: a case-report

**DOI:** 10.1186/s12890-020-01231-6

**Published:** 2020-07-31

**Authors:** Jake E. Cowen, James Stevenson, Madhusudan Paravasthu, James Darroch, Anu Jacob, Salaheddin Tueger, John R. Gosney, Anneliese Simons, Lisa G. Spencer, Eoin P. Judge

**Affiliations:** 1 Department of Respiratory Medicine, Aintree Chest Centre, Liverpool University Hospitals NHS Foundation Trust, Liverpool, L9 7AL UK; 2 Department of Pathology, Whiston Hospital, St Helen’s & Knowsley Teaching Hospitals NHS Trust, Merseyside, UK; 3 Department of Radiology, Aintree University Hospital, Liverpool University Hospitals NHS Foundation Trust, Liverpool, UK; 4 Department of Immunology, Royal Liverpool & Broadgreen Hospital, Liverpool University Hospitals NHS Foundation Trust, Liverpool, UK; 5 Department of Neurology, The Walton Centre, The Walton Centre NHS Foundation Trust, Liverpool, UK; 6 Department of Haematology, Countess of Chester Hospital, Countess of Chester Hospital NHS Foundation Trust, Chester, UK; 7 Department of Cellular Pathology, Liverpool University Hospitals NHS Foundation Trust, Liverpool, UK

**Keywords:** Common variable immunodeficiency, Granulomatous-lymphocytic interstitial lung disease, Sarcoidosis

## Abstract

**Background:**

Common variable immunodeficiency (CVID) is a group of heterogeneous primary immunodeficiencies characterised by a dysregulated and impaired immune response. In addition to an increased susceptibility to infection, it is also associated with noninfectious autoimmune and lymphoproliferative complications. CVID is rarely associated with neurological complications. Pulmonary involvement is more common, and patients can develop an interstitial lung disease known as granulomatous-lymphocytic interstitial lung disease (GLILD).

**Case presentation:**

A 50-year-old Caucasian female with a history of Evans syndrome (idiopathic thrombocytopaenic purpura and autoimmune haemolytic anaemia) and hypogammaglobulinaemia initially presented to the neurology clinic with marked cerebellar ataxia and headaches. Following extensive investigation (which included brain biopsy), she was diagnosed with neuro-sarcoidosis and her symptoms resolved following treatment with immunosuppressive therapy. Over the following 10 years, she was extensively investigated for recurrent pulmonary infections and abnormal radiological findings, which included pulmonary nodules, infiltrates and splenomegaly. Subsequently, she was referred to an immunology clinic, where immunoglobulin replacement treatment was started for what was ultimately considered to be CVID. Shortly afterwards, evaluation of her clinical, radiological and histological findings at a specialist interstitial lung disease clinic led to a diagnosis of GLILD.

**Conclusion:**

CVID is a condition which should be suspected in patients with immunodeficiency and recurrent infections. Concomitant autoimmune disorders such as haemolytic anaemia and immune thrombocytopenia may further support the diagnosis. As illustrated in this case, there is a rare association between CVID and inflammatory involvement of the neurological system. Respiratory physicians should also suspect CVID with associated GLILD in patients with apparent pulmonary granulomatous disease and recurrent infections. In addition, this case also highlights the challenge of diagnosing CVID and its associated features, and how the definitive exclusion of other pathologies such as malignancy, mycobacterial infection and lymphoma is required as part of this diagnostic process.

## Learning points

- A diagnosis of CVID can be difficult to make, but physicians from all specialties should suspect this diagnosis in patients with recurrent infections (especially respiratory infections) who have no obvious precipitating risk factors.
- CVID can be associated with systemic lymphocytic/granulomatous disease. CVID-associated neurological disease is rare but should be suspected in patients with a history of recurrent infections/immunodeficiency and lymphocytic/granulomatous changes on biopsy.
- GLILD should be suspected in patients with CVID and pulmonary disease. Diagnosis is based on a combination of compatible clinical, radiological and histological features.
- Exclusion of other pathologies such as malignancy, lymphoma, mycobacterial disease and sarcoidosis should be performed in all cases of suspected CVID-associated neurological disease and GLILD.

## Background

Common variable immunodeficiency (CVID) is a primary immune disorder whose principal manifestation is functional abnormality of the B cell lineage. The condition is characterised by hypoglobulinaemia (typically IgG and IgA) and susceptibility to bacterial infections, particularly polysaccharide-encapsulated organisms causing infections of the respiratory tract [1, 2]. CVID accounts for 90% of symptomatic antibody deficiencies and is known to present at any age [2]. The disorder is further associated with autoimmune conditions such as autoimmune haemolytic anaemia (AIHA), idiopathic thrombocytopenic purpura (ITP) and autoimmune neutropenia [2, 3].

Up to 22% of patients with CVID experience a systemic inflammatory/granulomatous form of the condition, characterised by lymphocytic and/or granulomatous infiltration of several organs, most commonly the lungs [4–6]. Granulomatous-lymphocytic interstitial lung disease (GLILD) is a distinct form of interstitial lung disease in patients with CVID. It is diagnosed based on specific clinical/radiological/histopathological features, following the exclusion of other pathologies [7].

Here we present the case of a patient who presented to the respiratory clinic with a long history of recurrent infections, hypogammaglobulinaemia (on treatment with intravenous immunoglobulin infusions), a preceding diagnosis of Evans syndrome, previously investigated neurological symptoms and a cerebellar lesion that was attributed to neurosarcoidosis, and chronic pulmonary disease which had been extensively investigated for malignancy and infection over several years. A unifying diagnosis of CVID with associated neurological disease, interstitial lung disease (GLILD) and autoimmune haemolytic disease was made, following careful consideration and multidisciplinary review of the clinical, radiological and histological findings in this case.

## Case presentation

A 50-year-old Caucasian female presented to the neurology clinic with marked cerebellar ataxia. Her medical history included Evans syndrome – a combination of idiopathic thrombocytopaenic purpura (ITP) and autoimmune haemolytic anaemia (AIHA) diagnosed in her 30s, hypogammaglobulinaemia with recurrent chest infections, and hypertension.

**Fig. 1:**
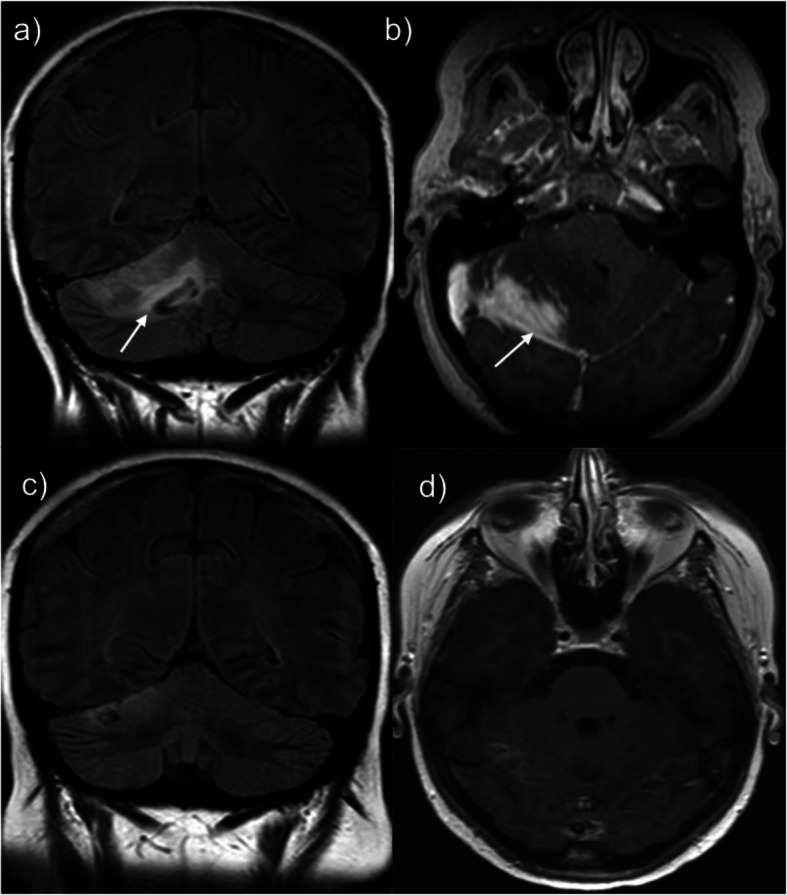
MRI brain imaging. MRI Brain showing high signal in the right cerebellar hemisphere. **a** Coronal FLAIR, arrow with a corresponding abnormal focal area of enhancement shown on the **b** Axial post-gadolinium images. Repeat MRI Brain after 3 months (while the patient was on treatment) shows almost complete resolution of these findings **c** Coronal FLAIR **d** Axial post-gadolinium

The patient underwent extensive investigation for these cerebellar symptoms at that time. CSF analysis was negative for infection, and relevant investigations for demyelinating conditions such as multiple sclerosis (including MRI spine) yielded negative results. MRI Brain showed an abnormal, wedge-shaped, bright signal intensity lesion in the right cerebellar hemisphere, with slight mass effect on the fourth ventricle. The area of abnormality extended through the grey and white matter (Fig. 1). A cerebellar biopsy was subsequently performed to exclude malignancy. This showed focal infiltration of both the cerebellum and meninges with macrophages and lymphocytes consistent with meningo-cerebellitis. No granulomata were seen, and the infiltrating lymphocytes were predominantly T-cells. Immunohistochemistry demonstrated no evidence of lymphoma. A chest x-ray (CXR) showed linear shadowing in the left midzone and nodular opacification in the right upper and lower zones. A unifying diagnosis of neurosarcoidosis was made on the basis of these clinico-radio-pathological findings.

The patient was treated with corticosteroids and methotrexate, and her symptoms and MRI brain abnormalities resolved within 3 months. This treatment was tapered off over a two-year period as her symptoms had resolved. Aside from intermittent headaches (which were treated with greater occipital nerves blocks), the patient did not experience any further neurological symptoms.

**Fig. 2:**
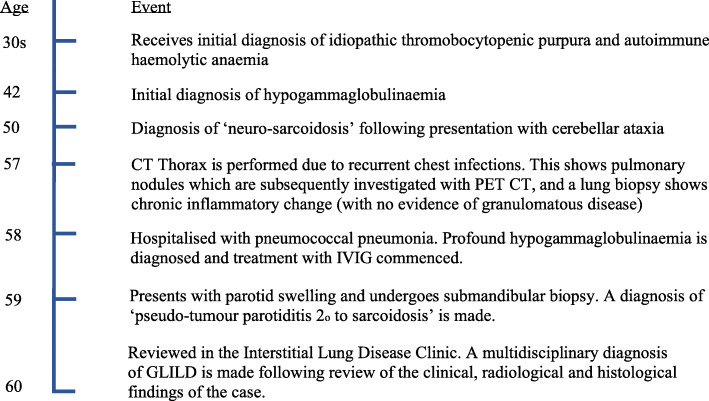
Timeline. Relevant medical history and clinical course

**Fig. 3:**
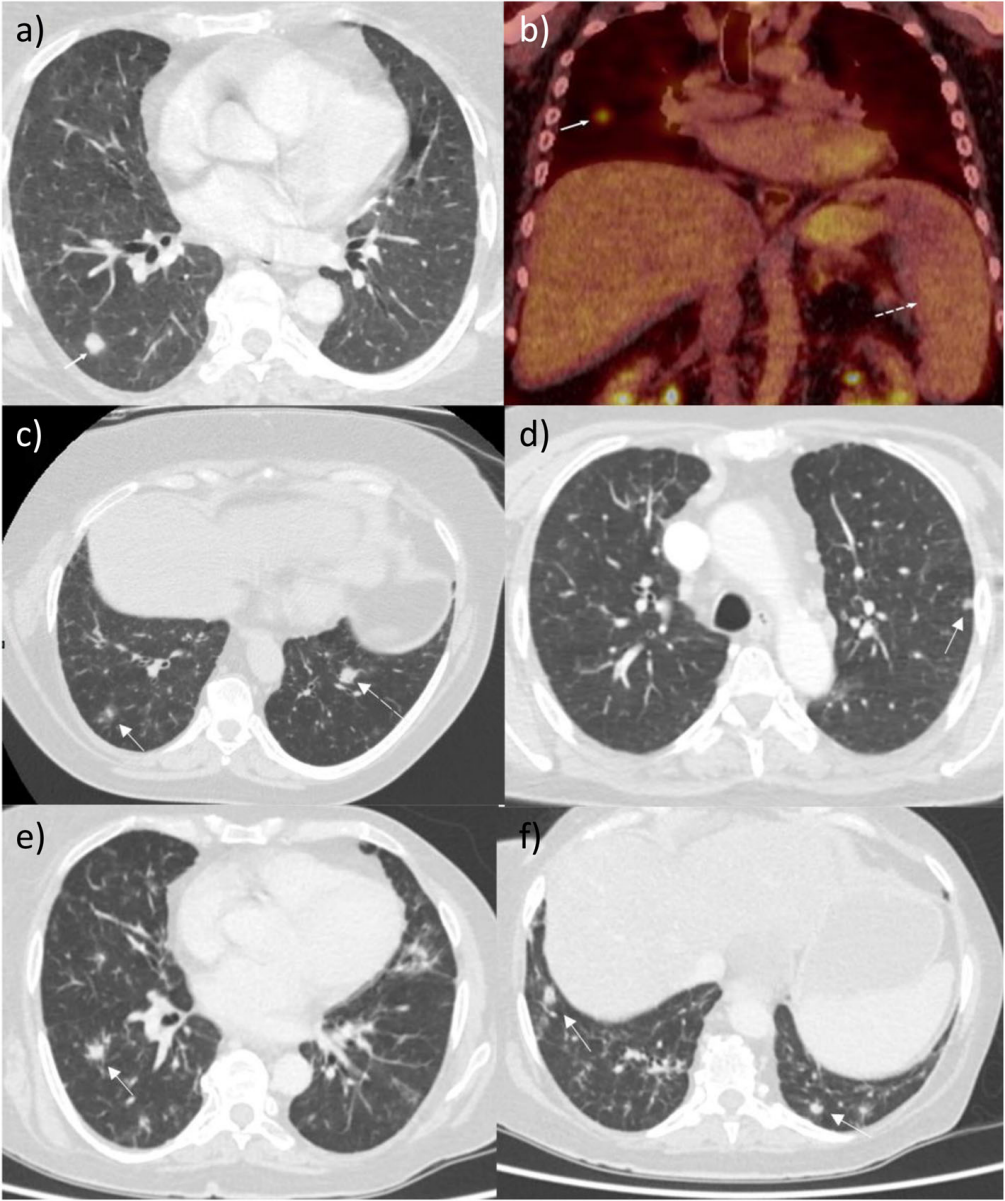
CT and PET imaging. **a** CT Thorax showing a right lower lobe pulmonary nodule (arrow, June 2016), **b** PET-CT showing the same right lower lobe pulmonary nodule (arrow) and splenomegaly (broken arrow). **c** New left lower lobe pulmonary nodule (broken arrow) and resolving right lower lobe nodule (arrow, July 2016). **d** New left lower lobe nodule (arrow, April 2018) **e** New right lower lobe nodule (arrow, Sept 2019) f) New lower lobe nodules (arrows, Sept 2019)

Approximately 7 years after her original presentation, she was noted to have pulmonary nodules on a CT thorax performed in a different hospital (Fig. 3). This scan was performed as she was experiencing symptoms suggestive of recurrent chest infections. These nodules were subsequently followed up (radiologically) over a 1-year-period, and a CT-guided lung biopsy of one nodule showed chronic inflammation (lymphocyte predominant) with no evidence of granulomatous disease. The patient was discharged from the respiratory service in that hospital at that point in time. During this period, she was also noted to have splenomegaly and haemolytic anaemia and was treated with immunosuppressive therapy (prednisolone with other agents e.g. rituximab at different points) for this by the haematology service (Fig. 2). She was also admitted to hospital around this time with pneumococcal pneumonia and sepsis and was found to be profoundly hypogammaglobulinaemic. Following assessment by the immunology service, she was commenced on immunoglobulin replacement therapy (initially intravenously, later converted to subcutaneous administration). A year later (aged 59) the patient developed parotid gland swelling and underwent sub-mandibular gland biopsy. The biopsy revealed smeared lymphocytes but no evidence of granulomata or malignancy. The patient was diagnosed with ‘pseudo-tumour parotiditis secondary to sarcoidosis’ and the swelling improved with steroid therapy.

Approximately 10 years after her initial presentation to the neurology clinic (aged 60), the patient was referred to the specialist Interstitial Lung Disease clinic for management of her sarcoidosis. At the time of clinic, she was relatively asymptomatic with respect to her chest, with no cough or exertional dyspnoea. There was no reported abdominal pain, chest pain, palpitations, reflux symptoms, night sweats or ocular symptoms. She described a flare of chest symptoms associated with splenomegaly some years previously when attempting to wean her steroids. The patient was a never smoker, with no known family history of pulmonary disease or immunodeficiency. She was a retired childcare worker who had no identifiable respiratory risk factors or previous inhalational exposures.

At time of her clinic appointment her medications were as follows: *Gammanorm* 8 g weekly subcutaneously, prednisolone 5 mg OD (slow tapering from 80 mg over several months), cyclosporine 200 mg OD, omeprazole 20 mg OD, cholecalciferol 20,000 IU twice weekly, alendronic acid 70 mg weekly, folic acid 5 mg OD, lisinopril 10 mg OD, fluoxetine 20 mg OD and ferrous sulphate 200 mg OD. She reported no known drug allergies.

On examination, her weight was 81 kg and her BMI 35. Her pulse rate was 84 bpm and regular, blood pressure 180/100 mmHg, temperature 36.8 °C and oxygen saturations 97% on room air. She was visibly cushingoid. She was clinically euvolaemic. Her cardiac examination was unremarkable. Pulmonary examination revealed some crackles in the right lower zone with no squawks or wheezes. Abdominal examination revealed an enlarged spleen 4 cm below the costal margin. The remainder of the clinical examination was unremarkable.

### Investigations & results

Blood tests demonstrated a mild anaemia (115 g/L) and thrombocytopenia (110 × 10^9^/L). Serum IgA (0.09 g/L) was low and serum IgG (6.4 g/L) was at the lower end of the normal range (patient was noted to be on immunoglobulin therapy at that time). There was no evidence of infection, while serum ACE (13 U/L), corrected calcium (2.19 mmol/L), liver enzymes, renal profile and autoimmune screen were all unremarkable. Spirometric lung volumes measured in the ILD clinic were within normal range, and stable over a 6-month period. Her FEV_1_ was measured as 2.23 l (114% predicted) and 2.18 l (114% predicted). Her FVC over the 6-month period was 2.55 l (112% predicted) and 2.65 l (113% predicted). FEV_1_/FVC was 85, and 84% at 6-months. Gas transfer and KCO were notably reduced at 48 and 64% predicted respectively.

Recent CXR showed no focal abnormalities, but review of previous CXRs showed fluctuating pulmonary nodular changes over a 10-year period.

Review of her CT thorax imaging (over a 3-year period) showed fluctuating bilateral parenchymal nodular changes (including fissural nodularity), and areas of ground glass opacification and reticular change. Stable sub-centimetre mediastinal adenopathy and massive splenomegaly (18 cm) were also noted. A PET CT performed during previous evaluation of her pulmonary nodularity showed a right lower lobe pulmonary nodule with low/moderate FDG avidity (Fig. 3).

Echocardiogram performed aged 60 was unremarkable, with normal left and right ventricular systolic function and no echocardiographic evidence of pulmonary hypertension.

**Fig. 4:**
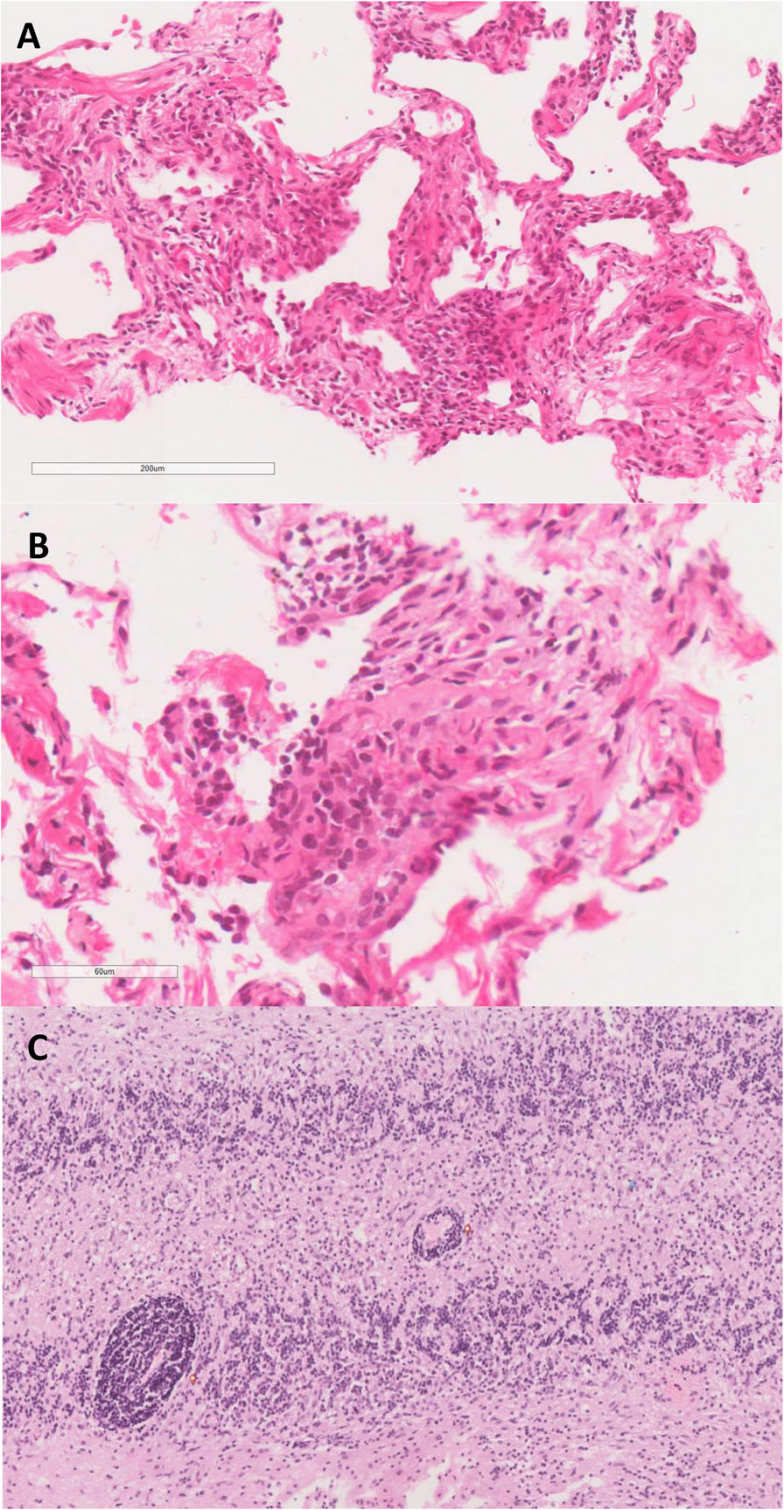
Pulmonary and cerebellar histology. Histopathological characteristics supporting a diagnosis of GLILD: **a** the core biopsy of pulmonary parenchyma showing a reticular pattern of fibrosis and interstitial lymphocytic infiltration (H & E, × 100 magnification); **b** at higher magnification, the lymphocytes can be seen to form aggregates giving a nodular appearance. On CD3 immunostaining these lymphocytes proved to be of predominantly T-cell lineage (H & E, × 400); **c** the cerebellar biopsy from the same patient 10 years previously showing lymphocytic infiltration (red dot) with a similar pattern to that seen in the lung biopsy (H & E, × 100)

Microscopy of fine needle aspirates, taken 3 years previously from a lung nodule, demonstrated a non-specific infiltrate, predominantly lymphoid in nature, with features consistent with a diagnosis of GLILD (Fig. 4).

### Diagnosis

A diagnosis of CVID with associated GLILD was made following multidisciplinary team review of the clinical, radiological and histological features of this case. Furthermore, re-review of her brain biopsy histology showed focal infiltration of lymphocytes and excluded other pathologies (lymphoma, mycobacterial disease), thereby supporting a retrospective diagnosis of CVID-associated neurological involvement.

## Discussion & Conclusions

CVID is a primary immune disorder primarily characterised by humoral immunodeficiency. Patients with the condition are characteristically hypogammaglobulinaemic, with increased susceptibility to bacterial infections [1, 2]. Splenomegaly may be seen in one third of patients, and previous studies suggest that the presence of AIHA, ITP or splenomegaly may identify patients at risk of developing interstitial pulmonary complications [2, 8]. No single underlying cause is known, and the condition may be polygenic in origin [9]. According to the European Society for Immunodeficiences (ESID), patients must fulfill at least one of the following diagnostic criteria in order for a diagnosis of CVID to be made:

i. Increased susceptibility to infection
ii. Autoimmune manifestations
iii. Granulomatous disease
iv. Unexplained polyclonal lymphoproliferation
v. Affected family member with antibody deficiency [10].

These clinical features must be combined with a marked decrease in IgG and IgA (more than 2 standard deviations below the normal levels for their age, measured at least twice) with or without low IgM levels, and patients must exhibit a poor antibody response to vaccines or low switched memory B cells. Secondary causes of hypogammaglobulinaemia must be excluded, disease onset must be after the 4th year of life, and profound T-cell deficiency should also be excluded [10].

The patient in this case fulfills the diagnostic criteria for CVID. Firstly, she experienced recurrent pulmonary infections over a number of years including an episode of confirmed pneumococcal pneumonia. Secondly, this patient had a diagnosis of immunodeficiency with a marked decrease in IgA and IgG levels and was being treated with regular IgG infusions at the time of presentation to the interstitial lung disease clinic. Alternative causes of hypogammaglobulinaemia and immunodeficiency such as haematological malignancy, HIV, or nutritional deficiency were fully investigated and excluded in this case. Thirdly, histological analysis demonstrated lymphocytic infiltration on cerebellar, submandibular and pulmonary biopsies, which is consistent with the histological features of CVID. Finally, the presence of splenomegaly and preceding diagnosis of Evans syndrome support the known association between autoimmune haematological disease and CVID.

In this case, the patient previously underwent a cerebellar biopsy that revealed ‘meningo-cerebellitis’, which was deemed compatible with a diagnosis of neurosarcoidosis in the context of her CXR findings at that time. In retrospect, this histological finding was probably a manifestation of CVID. Neurological associations with CVID are notably rare, and there are a very small number of reported cases in the literature [11–18]. Important differential diagnoses were excluded at the time. In this case, infection, primary malignancy and cerebral lymphoma were excluded following review of CSF and brain biopsy specimens. This patient’s CVID-associated neurological disease also responded well to immunosuppressive therapy, which is consistent with the findings of a previously published case report [13].

Pulmonary involvement is more common than neurological involvement in patients with CVID. Granulomatous-lymphocytic interstitial lung disease (GLILD) is a specific subtype of interstitial lung disease associated with CVID [4, 6, 7, 19, 20]. Until recently, there has been little consensus on the definition, diagnosis and management of GLILD. In 2017, a consensus document defined GLILD as a distinct clinico-radio-pathological ILD occurring in patients with CVID, associated with a lymphocytic infiltrate and/or granuloma in the lung, and in whom other conditions have been considered and where possible excluded [7]. Although a CT thorax is required to support a diagnosis of GLILD, there is no specific radiological feature which would be sufficient to confirm this diagnosis. Compatible CT thorax features include solid nodules, ground-glass opacities, hilar and/or mediastinal lymphadenopathy and reticulation [7, 21]. Rather, a surgical biopsy (preferably a video-assisted thoracic surgical (VATS) biopsy) is needed in combination with compatible clinical and radiological features to confirm the diagnosis [7]. Similarly, there is no specific histological feature of GLILD, but features of granulomatous inflammation, peribronchiolar lymphoid proliferation, interstitial lymphoid proliferation, and CD4+ T-Cell predominance are all felt to be characteristic of this condition [7].

Definitive exclusion of other potential pathologies such as mycobacterial infection, organizing pneumonia, malignancy and lymphoma is also a key consideration in the diagnosis of GLILD. Repeated sampling, Ziehl–Neelsen staining and culturing of respiratory specimens (sputum, tissue) excluded mycobacterial disease. A PET CT performed during evaluation of pulmonary nodules showed a nodule in the right lower lobe with low/moderate FDG avidity, and CT thorax scans showed fluctuating parenchymal nodular changes, ground glass opacification and reticular change – which are characteristic radiological features of GLILD. The patient also had a fine-needle aspirate (FNA) of one of the pulmonary nodules. Histological analysis of this tissue (in addition to the previous brain biopsy tissue) showed no evidence of malignancy, and immunohistochemical staining excluded lymphoma. Mycobacterial disease was also excluded, and there was no histological evidence of granulomas. Histological analysis did however demonstrate non-specific lymphoid infiltration (predominantly T cell type) which is considered to be a typical histological feature of GLILD. As previously mentioned, VATS lung biopsy is described as the biopsy modality of choice for diagnosing GLILD. However, this procedure is invasive and is associated with clinical risk, especially in patients with pre-existing severe lung disease [22]. In this case, following careful multidisciplinary team review of the clinical features and results of extensive investigation findings (radiological and histological), it was decided that a confident diagnosis of CVID with associated GLILD (and neurological involvement) could be made without the need for further tissue sampling (i.e. VATS biopsy).

Differentiating GLILD from sarcoidosis can be extremely challenging, as both conditions share a number of similar clinical, radiological and histological characteristics, such as non-specific pulmonary symptoms, splenomegaly, impaired lung function, interstitial infiltrates and pulmonary nodules on CT Thorax, and lymphocytic/granulomatous inflammation on lung biopsy. Indeed, in this case, the patient had been initially referred to the ILD clinic with suspected sarcoidosis. However, in contrast to sarcoidosis, which is often associated with hypergammaglobulinaemia, the patient’s history of primary immunodeficiency favoured a diagnosis of CVID-associated GLILD rather than sarcoidosis. Furthermore, specific radiological features, such as non-upper zone predominant interstitial changes and compatible biopsy features all favoured a diagnosis of CVID-associated GLILD.

This case also highlights the challenges facing clinicians in diagnosing rare diseases such as CVID and its systemic manifestations, such as neurological disease and GLILD. Indeed, CVID is associated with a diagnostic delay of over 15 years in 20% of patients [2, 20, 23, 24]. In this case, there was a period of 10 years between the patient’s initial presentation and the final multidisciplinary diagnosis. In addition, this case also emphasises the importance of multidisciplinary team review and cross-specialty collaboration in the diagnosis and management of rare multiorgan diseases such as CVID.

## Data Availability

All data is available in the manuscript.

## Abbreviations

CXR: Chest X-ray
CT: Computerised tomography
CVID: Common variable immunodeficiency
DVT: Deep vein thrombosis
FEV1: Forced expiratory volume during first second of expiration
FVC: Forced vital capacity
GLILD: Granulomatous-lymphocytic interstitial lung disease
HRCT: High resolution computerised tomography scan
ILD: Interstitial lung disease
KCO: Transfer coefficient of the lung for carbon monoxide
OD: Once daily
VATS: Video-assisted thoracic surgical biopsy
